# COVID-19–Related Hospitalization Rates and Severe Outcomes Among Veterans From 5 Veterans Affairs Medical Centers: Hospital-Based Surveillance Study

**DOI:** 10.2196/24502

**Published:** 2021-01-22

**Authors:** Cristina V Cardemil, Rebecca Dahl, Mila M Prill, Jordan Cates, Sheldon Brown, Adrienne Perea, Vincent Marconi, LaSara Bell, Maria C Rodriguez-Barradas, Gilberto Rivera-Dominguez, David Beenhouwer, Aleksandra Poteshkina, Mark Holodniy, Cynthia Lucero-Obusan, Neha Balachandran, Aron J Hall, Lindsay Kim, Gayle Langley

**Affiliations:** 1 Centers for Disease Control and Prevention Atlanta, GA United States; 2 United States Public Health Service Rockville, MD United States; 3 James J. Peters VA Medical Center New York, NY United States; 4 Icahn School of Medicine at Mt. Sinai New York, NY United States; 5 Atlanta VA Medical Center Atlanta, GA United States; 6 Emory University School of Medicine Atlanta, GA United States; 7 Rollins School of Public Health Emory University Atlanta, GA United States; 8 Infectious Diseases Section Department of Medicine Baylor College of Medicine Houston, TX United States; 9 Michael E. DeBakey VA Medical Center Houston, TX United States; 10 VA Greater Los Angeles Healthcare System Los Angeles, CA United States; 11 David Geffen School of Medicine at UCLA Los Angeles, CA United States; 12 Public Health Surveillance and Research, Department of Veterans Affairs Washington, DC United States; 13 VA Palo Alto Health Care System Palo Alto, CA United States; 14 Division of Infectious Diseases and Geographic Medicine Stanford University Stanford, CA United States

**Keywords:** veteran, COVID-19, hospitalization, outcome, fatality, mortality, prevention, at-risk

## Abstract

**Background:**

COVID-19 has disproportionately affected older adults and certain racial and ethnic groups in the United States. Data quantifying the disease burden, as well as describing clinical outcomes during hospitalization among these groups, are needed.

**Objective:**

We aimed to describe interim COVID-19 hospitalization rates and severe clinical outcomes by age group and race and ethnicity among US veterans by using a multisite surveillance network.

**Methods:**

We implemented a multisite COVID-19 surveillance platform in 5 Veterans Affairs Medical Centers located in Atlanta, Bronx, Houston, Palo Alto, and Los Angeles, collectively serving more than 396,000 patients annually. From February 27 to July 17, 2020, we actively identified inpatient cases with COVID-19 by screening admitted patients and reviewing their laboratory test results. We then manually abstracted the patients' medical charts for demographics, underlying medical conditions, and clinical outcomes. Furthermore, we calculated hospitalization incidence and incidence rate ratios, as well as relative risk for invasive mechanical ventilation, intensive care unit admission, and case fatality rate after adjusting for age, race and ethnicity, and underlying medical conditions.

**Results:**

We identified 621 laboratory-confirmed, hospitalized COVID-19 cases. The median age of the patients was 70 years, with 65.7% (408/621) aged ≥65 years and 94% (584/621) male. Most COVID-19 diagnoses were among non-Hispanic Black (325/621, 52.3%) veterans, followed by non-Hispanic White (153/621, 24.6%) and Hispanic or Latino (112/621, 18%) veterans. Hospitalization rates were the highest among veterans who were ≥85 years old, Hispanic or Latino, and non-Hispanic Black (430, 317, and 298 per 100,000, respectively). Veterans aged ≥85 years had a 14-fold increased rate of hospitalization compared with those aged 18-29 years (95% CI: 5.7-34.6), whereas Hispanic or Latino and Black veterans had a 4.6- and 4.2-fold increased rate of hospitalization, respectively, compared with non-Hispanic White veterans (95% CI: 3.6-5.9). Overall, 11.6% (72/621) of the patients required invasive mechanical ventilation, 26.6% (165/621) were admitted to the intensive care unit, and 16.9% (105/621) died in the hospital. The adjusted relative risk for invasive mechanical ventilation and admission to the intensive care unit did not differ by age group or race and ethnicity, but veterans aged ≥65 years had a 4.5-fold increased risk of death while hospitalized with COVID-19 compared with those aged <65 years (95% CI: 2.4-8.6).

**Conclusions:**

COVID-19 surveillance at the 5 Veterans Affairs Medical Centers across the United States demonstrated higher hospitalization rates and severe outcomes among older veterans, as well as higher hospitalization rates among Hispanic or Latino and non-Hispanic Black veterans than among non-Hispanic White veterans. These findings highlight the need for targeted prevention and timely treatment for veterans, with special attention to older aged, Hispanic or Latino, and non-Hispanic Black veterans.

## Introduction

COVID-19 has been found to be more prevalent in older adults, men, and those with certain underlying comorbidities, thereby resulting in higher rates of hospitalization and deaths among these groups [1,2]. The COVID-19 burden has also disproportionately affected certain racial and ethnic groups in the United States, including non-Hispanic Black, Hispanic or Latino, and American Indian or Alaska Native persons [3].

Over 9 million veterans (defined here as former members of the armed forces) are enrolled in the Veterans Affairs (VA)–integrated health care program [4]. These US veterans constitute a population that is older, is predominantly male, has a high rate of chronic medical conditions, and has a higher representation from certain racial and ethnic groups compared with the general population. We built a COVID-19 surveillance system on the infrastructure of SUPERNOVA (Surveillance Platform for Enteric and Respiratory Infectious Organisms in the Veterans Affairs population), a population-based platform that captures information on cases of veterans with acute gastroenteritis and acute respiratory illness [5] from 5 VA Medical Centers (VAMCs) located in Atlanta, Georgia; Bronx, New York; Houston, Texas; Los Angeles, California; and Palo Alto, California. This surveillance system helps understand the epidemiology, hospitalization rates, and underlying medical conditions among these US veterans. There are limited studies describing the impact of COVID-19 on this specific population, but recently published articles indicate that although higher rates of hospitalization and severe outcomes are not observed among Black and Hispanic veterans, they are more likely to test positive for COVID-19 and have higher risks for sepsis and respiratory, neurologic, and renal in-hospital complications than White veterans [6-9]. The objective of this study was to describe the interim COVID-19 hospitalization rates, severe outcomes, and prevalence of underlying medical conditions by age group and race and ethnicity among veterans in this network.

## Methods

### Study Population

SUPERNOVA sites served 396,280 unique veterans in outpatient and inpatient settings in the fiscal year 2019 (Atlanta: n=118,258; Bronx: n=24,116; Houston: n=109,890; Los Angeles: n=82,574; and Palo Alto: n=61,442). The veteran catchment population at these sites was 32% (126,188/396,280) non-Hispanic Black, 47% (186,174/396,280) non-Hispanic White, and 11% (42,098/396,280) Hispanic or Latino. The overall median age of the veterans in the catchment population was 64 years, and 87% (342,944/396,280) of all veterans were male.

### Screening and Data Abstraction

To identify eligible patients for COVID-19 surveillance, trained research personnel screened admission logs and diagnoses [5], lists of patients being tested for COVID-19, and the results from respiratory specimens submitted for testing. Patients were eligible for inclusion in this study if they were admitted to the VAMC and had a positive molecular test for SARS-CoV-2 within 14 days prior to admission or during hospitalization. COVID-19 testing was ordered by clinicians either prior to or during hospitalization, and tests were conducted at the hospital or at public or commercial laboratories serving each VAMC. Data were manually summarized from patients’ electronic medical charts. Research personnel reviewed the patient’s entire hospitalization course such as admission and discharge notes; International Classification of Diseases, Tenth Revision, Clinical Modification (ICD-10-CM) discharge codes; and problem lists to identify patients with any underlying medical conditions on a prespecified list. The complete list of variables for abstraction included over 140 underlying medical conditions grouped into 10 categories. Therefore, for rapid abstraction and analyses, the data abstraction for this report was intentionally limited to the underlying medical conditions (ie, chronic kidney disease, chronic obstructive pulmonary disease (COPD), coronary artery disease, heart failure, hypertension, diabetes, and obesity) that had been identified from surveillance early during the pandemic as being more prevalent among hospitalized COVID-19 patients [10]. This report presents data analyzed for patients admitted from February 27 to July 17, 2020, and their clinical outcomes available through July 31, 2020.

### Statistical Analysis

Hospitalization incidence per 100,000 person-years among veterans was calculated based on previously published methods by using the catchment population of unique individuals served at each facility [11]. Severe clinical outcomes were defined as patients requiring invasive mechanical ventilation, admission to an intensive care unit, or deaths among hospitalized patients. Binomial regression was used to generate incidence rate ratios and 95% CIs for hospitalization rates, and relative risks and 95% CI for severe outcomes. All models were adjusted for age and race and ethnicity; outcome models were also adjusted for the number of underlying medical conditions.

Case fatality rate (CFR) was calculated as a 14-day moving average. We plotted the distribution of cases and compared the CFR over 2 time periods: one early during the pandemic and one later in the summer months. Patients who were still hospitalized were excluded from the analysis. Differences between groups and time periods were compared by chi-square tests and considered significant if *P*<.05.

### Ethical Review

This study was reviewed, informed consent was waived, and a final approval was obtained by 5 VA sites, research and development committees, and the Centers for Disease Control and Prevention institutional review board(s).

## Results

**Figure 1:**
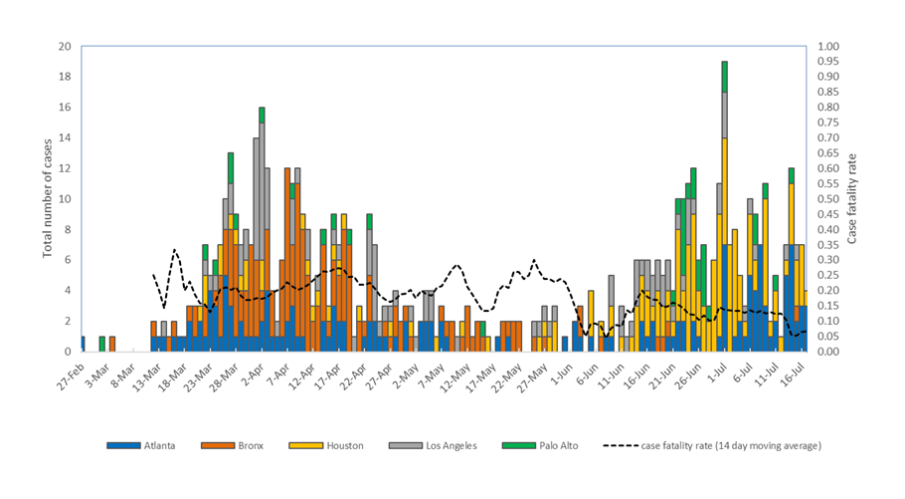
Epidemic curve of hospitalized COVID-19 cases and case fatality rate for US veterans at 5 Veterans Affairs Medical Centers by hospital admission date, from February 27 to July 17, 2020. Case fatality rate represents a 14-day moving average. Rate suppressed prior to March 12, 2020, due to low case counts (n≤3).

From February 27 through July 17, 2020, a total of 621 laboratory-confirmed, hospitalized COVID-19 cases were identified at the 5 VAMCs. As of July 31, 2020, 27 of the 621 (4.3%) patients continued to remain hospitalized. These cases initially peaked in late March and April 2020, before declining and then plateauing in May and June, and then increased again in the latter half of June and early July, with the highest peak in new hospital admissions recorded on July 1, 2020 (Figure 1). The CFR among patients whose hospitalization was completed was higher from February 27 to May 31 (75/343, 21.9%) than from June 1 to July 17 (30/251, 12%; *P*<.01).

The Bronx VAMC had the highest number of hospitalized veterans diagnosed with COVID-19 (166/621, 27%), followed by Atlanta (157/621, 25%), Houston (156/621, 25%), Los Angeles (105/621, 17%), and Palo Alto (37/621, 6%). Across all VAMCs, the patients’ median age was 70 years, with 66% (408/621) of the patients aged ≥65 years and 94% (584/621), male. The highest percentage of hospitalizations due to confirmed COVID-19 diagnoses were among non-Hispanic Black veterans (325/621, 52.3%), followed by non-Hispanic White (153/621, 24.6%), Hispanic or Latino (112/621, 18%), non-Hispanic Asian (9/621, 2%), non-Hispanic American Indian or Alaskan Native (5/621, 1%), and non-Hispanic Native Hawaiian or Pacific Islander (2/621, 0.3%) veterans. About 2.4% (15/621) of all veterans had an unknown race or ethnicity.

The overall hospitalization rate was 205.7 per 100,000 population (Table 1). Hospitalization rates were the highest among veterans who were aged ≥85 years, Hispanic or Latino, and non-Hispanic Black (430, 317, and 298 per 100,000, respectively). Veterans aged ≥85 years had a 14-fold increased rate of hospitalization relative to those aged 18-29 years (95% CI: 5.7-34.6), after adjusting for race and ethnicity. Compared with non-Hispanic White veterans, Hispanic or Latino veterans had a 4.6-fold increased rate of hospitalization (95% CI: 3.6-5.9), and non-Hispanic Black veterans had a 4.2-fold increased rate of hospitalization (95% CI: 3.4-5.1), after adjusting for age.

**Table 1:** Distribution characteristics, incidence rates, and adjusted incidence rate ratios of US veterans hospitalized with COVID-19—overall, by age, and by race and ethnicity, at 5 Veterans Affairs Medical Centers from February 27 to July 17, 2020.

Variable			Overall distribution of characteristics, n (%)		Hospitalization incidence rates, per 100,000		Adjusted incidence rate ratios of hospitalization (95% CI)^a^
Overall			621 (100)		205.7		N/A^b^
**Age range (years)**							
	18-29	5 (0.8)		48.4		ref^c^	
	30-39	21 (3.4)		61.7		1.31 (0.50-3.48)	
	40-49	37 (6)		121.2		2.25 (0.88-5.74)	
	50-64	150 (24.2)		190.9		3.68 (1.51-9.00)	
	65-74	218 (35.1)		238.6		5.89 (2.42-14.32)	
	75-84	107 (17.2)		282.7		8.16 (3.32-20.06)	
	≥85	83 (13.4)		429.7		14.00 (5.66-34.63)	
**Age group (years)**							
	<65	213 (34.3)		138.8		ref	
	≥65	408 (65.7)		274.7		2.65 (2.23-3.15)	
**Race and ethnicity**							
	Non-Hispanic White	153 (24.6)		96.1		ref	
	Hispanic or Latino	112 (18)		317.3		4.57 (3.58-5.85)	
	Non-Hispanic Black	325 (52.3)		297.9		4.18 (3.43-5.10)	

^a^Incidence rate ratio by age group is adjusted for race and ethnicity; incidence rate ratio by race and ethnicity is adjusted for age.

^b^N/A: not applicable.

^c^ref: reference group.

Overall, 11.6% (72/621) of the patients required invasive mechanical ventilation, 26.6% (165/621) were admitted to the intensive care unit (ICU), and 16.9% (105/621) died while hospitalized (Table 2). Although the adjusted relative risk for invasive mechanical ventilation and ICU admission was not significantly different by age group or by race and ethnicity, an increased risk of death was observed among older adults. Notably, veterans who were aged ≥65 years had a 4.5-fold increased risk of death while hospitalized with COVID-19 compared with those aged <65 years (95% CI: 2.4-8.6), after adjusting for race and ethnicity and the number of underlying medical conditions. Additionally, most deaths were among those who were aged ≥65 years (94/105, 89.5%), and veterans who were aged ≥85 years had the highest CFR (33/105, 39.8%).

**Table 2:** Prevalence and adjusted relative risk of invasive mechanical ventilation, intensive care unit (ICU) admission, and deaths among US veterans hospitalized with COVID-19—overall, by age, and by race and ethnicity, at 5 Veterans Affairs Medical Centers from February 27 to July 17, 2020.

Variable		Prevalence of invasive mechanical ventilation, n (%)	Adjusted relative risk for invasive mechanical ventilation (95% CI)^a^	Prevalence of ICU admission, n (%)	Adjusted relative risk for ICU admission (95% CI)^a^	Case fatality, n (%)	Adjusted relative risk for case fatality (95% CI)^a^	
Overall		72 (11.6)	N/A^b^	165 (26.6)	N/A	105 (16.9)	N/A	
**Age range (years)**								
	18-29	0 (0)	—^c^	1 (20)	—	0 (0)	—	
	30-39	1 (4.8)	—	4 (19)	—	0 (0)	—	
	40-49	2 (5.4)	—	3 (8.1)	—	1 (2.7)	—	
	50-64	14 (9.3)	ref^d^	37 (24.7)	ref^d^	10 (6.7)	ref^d^	
	65-74	31 (14.2)	1.51 (0.81-2.83)	62 (28.4)	1.12 (0.77-1.60)	36 (16.5)	2.65 (1.31-5.37)	
	75-84	14 (13.1)	1.10 (0.50-2.42)	39 (36.4)	1.34 (0.90-2.00)	25 (23.4)	3.20 (1.52-6.74)	
	≥85	10 (12)	1.12 (0.48-2.59)	19 (22.9)	0.88 (0.53-1.47)	33 (39.8)	6.28 (3.15-12.54)	
**Age group (years)**								
	<65	17 (8)	ref^e^	45 (21.1)	ref^e^	11 (5.2)	ref^e^	
	≥65	55 (13.5)	1.48 (0.85-2.59)	120 (29.4)	1.24 (0.90-1.71)	94 (23)	4.50 (2.37-8.56)	
**Race and ethnicity**								
	Non-Hispanic White	16 (10.5)	ref	47 (30.7)	ref	33 (21.6)	ref	
	Hispanic or Latino	14 (12.5)	1.16 (0.66-2.03)	25 (22.3)	0.90 (0.66-1.21)	19 (17)	0.86 (0.59-1.26)	
	non-Hispanic Black	35 (10.8)	1.38 (0.70-2.69)	84 (25.8)	0.79 (0.52-1.21)	46 (14.2)	0.90 (0.56-1.45)	

^a^Relative risk by age group is adjusted for race and ethnicity and the number of underlying medical conditions (chronic kidney disease, chronic obstructive pulmonary disease, coronary artery disease, heart failure, hypertension, diabetes, and obesity); relative risk by race and ethnicity is adjusted for age and underlying medical conditions.

^b^N/A: not applicable.

^c^Due to the small number of cases in the younger age groups, only veterans aged ≥50 years were included in these models.

^d^Veterans aged 50-64 years were the reference group for veterans aged 65-74 years, 75-84 years, and ≥85 years.

^e^For the models comparing veterans aged <65 years with ≥65 years, all cases and age groups were included (≥18 years) and combined into the 2 age groups (ie, 18-64 and ≥65 years old). Veterans aged 18-64 years old served as the reference group.

Most patients had at least one of the 7 underlying medical conditions (547/621, 88.1%) (Table 3). By age group, veterans who were aged ≥65 years had a higher prevalence of the following underlying medical conditions than those aged <65 years: chronic kidney disease (104/408, 25.5% vs 30/213, 14.1%; *P*=.001), COPD (76/408, 18.6% vs 14/213, 6.6%; *P*<.001), coronary artery disease (103/408, 25.2% vs 17/213, 8%; *P*<.001), heart failure (78/408, 19.1% vs 11/213, 5.2%; *P*<.001), hypertension (322/408, 78.9% vs 120/213, 56.3%; *P*<.001), and diabetes (218/408, 53.4% vs 88/213, 41.3%; *P*=.004). Veterans aged <65 years had a higher prevalence of obesity than those aged ≥65 years (93/213, 43.7% vs 84/408, 20.6%; *P*<.001). By race and ethnicity, non-Hispanic Black veterans (78/325, 24%) had a higher prevalence of chronic kidney disease than Hispanic or Latino (20/112, 17.9%) and non-Hispanic White veterans (29/153, 19%; *P*=.26), whereas non-Hispanic White veterans had a higher prevalence of COPD (non-Hispanic White: 40/153, 26.1%; non-Hispanic Black: 32/325, 9.9%; Hispanic or Latino: 15/112, 13.4%; *P*<.001).

**Table 3:** Prevalence of underlying medical conditions among US veterans hospitalized with COVID-19—overall, by age, and by race and ethnicity, at 5 Veterans Affairs Medical Centers from February 27 to July 17, 2020.

Underlying medical conditions	Overall (n=621), n (%)	Age group				Race and ethnicity				
		<65 years (n=213), n (%)	≥65 years (n=408), n (%)	*P* value	Hispanic or Latino (n=112), n (%)		Non-Hispanic Black (n=325), n (%)	Non-Hispanic White (n=153), n (%)	*P* value^a^	
Any underlying medical condition^b^	547 (88.1)	166 (77.9)	381 (93.4)	<.001	91 (81.3)		287 (88.3)	140 (91.5)	.04	
Chronic kidney disease^c^	134 (21.6)	30 (14.1)	104 (25.5)	.001	20 (17.9)		78 (24)	29 (19)	.26	
COPD^d^/emphysema	90 (14.5)	14 (6.6)	76 (18.6)	<.001	15 (13.4)		32 (9.8)	40 (26.1)	<.001	
Coronary artery disease	120 (19.3)	17 (8)	103 (25.2)	<.001	27 (24.1)		50 (15.4)	34 (22.2)	.06	
Heart failure	89 (14.3)	11 (5.2)	78 (19.1)	<.001	17 (15.2)		44 (13.5)	25 (16.3)	.71	
Hypertension^e^	442 (71.2)	120 (56.3)	322 (78.9)	<.001	74 (66.1)		233 (71.7)	110 (71.9)	.49	
Diabetes	306 (49.3)	88 (41.3)	218 (53.4)	.004	56 (50)		166 (51.1)	71 (46.4)	.63	
Obesity^e^	177 (28.5)	93 (43.7)	84 (20.6)	<.001	32 (28.6)		97 (29.8)	40 (26.1)	.70	

^a^Due to the small number of cases in the other race and ethnicity groups (non-Hispanic Asian, non-Hispanic American Indian or Alaskan Native, and non-Hispanic Native Hawaiian or Pacific Islander), comparisons were limited to the 3 race and ethnicity groups listed in this table.

^b^Any of the 7 underlying medical conditions listed in this table.

^c^Chronic kidney disease or chronic renal insufficiency.

^d^COPD: chronic obstructive pulmonary disease.

^e^Presence of hypertension and obesity were not determined based on blood pressure or height and weight measurements during the current hospitalization but were noted as part of the patient’s underlying medical history after review of clinical admission and discharge notes as well as discharge codes of the International Classification of Diseases, Tenth Revision, Clinical Modification.

## Discussion

The burden of COVID-19 hospitalizations at these 5 VAMCs was the highest among older, Hispanic or Latino, and non-Hispanic Black veterans. Veterans aged ≥50 years had a higher rate of hospitalization than those aged 18-29 years, and Hispanic or Latino and non-Hispanic Black veterans had a 4.6- and 4.2-fold higher rate of hospitalization, respectively, than non-Hispanic White veterans. More severe outcomes were noted among older adults, with 9 out of every 10 deaths reported among those who were aged ≥65 years. Moreover, these older veterans also had a 4.5-fold increased risk of death following hospitalization with COVID-19 as compared to those aged <65 years. These findings highlight the need for targeted prevention, control and treatment efforts for veterans, with special attention to increasing age and race and ethnicity (ie, Hispanic or Latino and non-Hispanic Black).

Our COVID-19 surveillance data demonstrated that Hispanic or Latino and non-Hispanic Black veterans were hospitalized for COVID-19 at higher rates than were non-Hispanic White veterans, which is consistent with the findings of studies in the United States and other countries [1,3,12]. Despite the higher hospitalization rates in these racial and ethnic groups, our findings did not show differences in severe outcomes by race and ethnicity, as measured by the need for invasive mechanical ventilation, ICU admission, or in-hospital deaths due to COVID-19. However, recently published data indicates a disproportionate burden of certain in-hospital complications among US veterans of racial and ethnic minority groups [9]. Therefore, there is an urgent need to understand sociodemographic and economic factors, access to care, living conditions, prior medical history, and occupational groups and exposures [13] that may be contributors to disparities in COVID-19 hospitalization rates as well as the short- and long-term clinical course after infection among US veteran minority populations.

Similar to nationwide death certificate trends, we observed a decline in the CFR since the start of the pandemic [10]. Continued surveillance will help track whether these trends persist and understand possible explanations for such trends, which may include earlier and wider access to testing and treatment, increased knowledge and availability of interventions, and possible differences in the demographics of those infected. The higher death rates observed among older individuals in this study was also noted in previous studies from China, Italy, and the United States [2,14-17]. Another surveillance system in the United States, Coronavirus Disease 2019-associated Hospitalization Surveillance Network (COVID-NET), has demonstrated a similar increase in hospitalization incidence and death with increasing age [17], indicating the need for continued prevention efforts for older adults.

Nearly all patients (88.1%) in this cohort had an underlying medical condition, and veterans aged ≥65 years had higher prevalence of several key comorbidities. Many studies have indicated the heightened risk of both older age and specific underlying medical conditions (eg, chronic kidney disease, COPD, serious heart conditions, and diabetes) for severe COVID-19 outcomes, including hospitalization, ICU admission, mechanical ventilation, and death [17,18]. Veterans who use VA health care are older and more likely to be diagnosed with multiple health conditions than the general population [19], making this an important group to monitor for severe outcomes resulting from COVID-19.

This study has limitations. First, early during the pandemic, limited COVID-19 testing was conducted nationwide, and this may have led to an under-ascertainment of patients with COVID-19. Second, although prior studies have indicated that 91% of veterans enrolled in VA healthcare in these 5 site catchment areas use their local VAMCs for care [5], veterans hospitalized elsewhere would not have been captured in our surveillance, thereby resulting in underestimation of hospitalization rates. Third, as the number of cases in younger age groups was much smaller than in older adults, we were not able to calculate relative statistics for severe clinical outcomes in veterans aged 18-49 years. Finally, veterans are predominantly male; therefore, these results may not be representative of all US adults.

In conclusion, multisite COVID-19 surveillance conducted at 5 VAMCs across the United States from February 27 to July 17, 2020, demonstrated higher hospitalization rates and more deaths among older veterans as well as higher hospitalization rates among Hispanic or Latino and non-Hispanic Black veterans than among non-Hispanic White veterans. To prevent contracting and spreading COVID-19, veterans and persons living with them should follow recommendations such as practicing social and physical distancing, regularly washing hands, and wearing masks, including inside the house. From a systems-level perspective, policies and procedures that focus on veterans, including timely and high-quality care as well as access to therapeutics and future vaccines, will help protect this population and reduce morbidity and mortality therein. Ongoing data collection at these sites will aid in further characterizing the clinical characteristics of COVID-19, better understanding the risk factors and management of hospitalized patients, characterization of the immune response to SARS-CoV-2 infection through collection of patients’ serum samples, and description of long-term outcomes following hospitalization.

## Abbreviations

CFR: case fatality rate
COPD: chronic obstructive pulmonary disease
ICU: intensive care unit
SUPERNOVA: Surveillance Platform for Enteric and Respiratory Infectious Organisms in the Veterans Affairs population
VA: Veterans Affairs
VAMC: Veterans Affairs Medical Centers
